# Efficacy of radiotherapy for the treatment of cystic echinococcosis in naturally infected sheep

**DOI:** 10.1186/s40249-017-0301-7

**Published:** 2017-05-03

**Authors:** Rui Mao, Wen-Bao Zhang, Hong-Zhi Qi, Tao Jiang, Ge Wu, Peng-Fei Lu, Abudula Ainiwaer, Ge Shang, Lin Xu, Jie Hao, Xi Shou, Hai-Tao Li, Jun Li, Song-An Zhang, Yong-Xing Bao, Hao Wen

**Affiliations:** 1 Department of Radiation Oncology, The First Affiliated Hospital of Xinjiang Medical University, Urumqi, 830054 Xinjiang China; 2 State Key Laboratory Incubation Base for Xinjiang Major Diseases Research and Xinjiang Key Laboratory of Echinococcosis, The First Affiliated Hospital of Xinjiang Medical University, Urumqi, 830054 Xinjiang China

**Keywords:** Radiotherapy, Cystic echinococcosis, *Echinococcus granulosus*, Effectiveness, Safety, Treatment, Sheep, China

## Abstract

**Background:**

Radiotherapy is commonly used to treat cancers. To date, there has been no study focusing on the effects of radiotherapy on hydatid disease in large animals. In this study, we aim to investigate the efficiency and safety of radiotherapy for treating hydatid disease caused by *Echinococcus granulosus* in naturally infected sheep.

**Methods:**

Ultrasound was used to screen naturally infected sheep in an echinococcosis endemic area in Xinjiang, China. A computer tomography (CT) scan confirmed the presence of hydatid cysts. Twenty sheep naturally infected with *E. granulosus* in the liver and/or lungs were randomly assigned into four groups receiving no irradiation, or X-ray irradiation of low (30 Gy), medium (45 Gy), and high dose (60 Gy), respectively. After three months of radiotherapy, a CT scan was performed to measure the changes in the cysts. The hepatic parasite cysts and host tissues were collected for histology and gene expression analysis.

**Results:**

In the animals subject to irradiation, no significant differences were observed in their appetite, daily activities, and weight before and after radiotherapy. Severe calcification was noticed in the cysts subject to a high dose of radiation compared with the groups subject to low and medium doses. Hematoxylin and eosin staining showed that irradiation contributed to the damage of the cyst structure and nucleus in the germinal layers. Quantitative PCR demonstrated that expression of TPX and HSP70 significantly decreased in a dose-dependent manner (*P* < 0.05). The expression of the EPC1 decreased in the medium- and high-dose groups compared with the low-dose group (*P* < 0.05). Meanwhile, the expression of radiation-related apoptosis genes caspase-3 and Gadd45 decreased with an increase in the irradiation dose.

**Conclusions:**

Radiotherapy is an option with satisfactory efficiency and safety for treating cystic echinococcosis in sheep with partial response or stable disease at month 3. In future, inhibition of cystic activity using radiotherapy may serve as a new regimen for treating hydatid disease.

**Electronic supplementary material:**

The online version of this article (doi:10.1186/s40249-017-0301-7) contains supplementary material, which is available to authorized users.

## Multilingual abstracts

Please see Additional file 1 for translations of the abstract into the five official working languages of the United Nations.

## Background

Cystic echinococcosis (CE), a zoonotic disease caused by *Echinococcus granulosus* [1–3], has become a major public health and economic problem worldwide. An epidemiology survey conducted in 2011 showed that more than 380 000 people were diagnosed with CE in China [4], but treatment remains a challenge. Currently, surgery is the preferred treatment for CE, however, a higher incidence of relapse has been reported. Albendazole has been widely considered as the first-line drug for treating CE, however, the efficiency of chemotherapy is not satisfactory as the agent concentration in the hydatid cysts was low [5]. Therefore, it is urgent to develop a new regimen for treating CE.

Radiotherapy may represent an alternative treatment modality, but there is no adequate evidence for it as yet. Up until now, very few studies have been carried out to evaluate the efficiency of radiotherapy for treating such diseases [6–8]. In 2013, Ulger et al. [6] reported that one patient with hydatid cysts in the chest wall, who showed no response to surgery and drugs, achieved local control and pain relief after radiotherapy. Previous studies conducted by us showed that X-ray is lethal for alveolar hydatid disease as it can increase its mortality rate and inhibit cyst growth in vivo (gerbil) or in vitro [7, 8].

To date, there has been no study that has investigated the effects of radiotherapy on hydatid disease in large animals worldwide. In this study, we aim to determine the efficiency and safety of radiotherapy to treat CE in naturally infected sheep.

## Methods

### Study subjects

Twenty naturally infected female sheep (aged 3 – 5 years) with liver cystic hydatid disease obtained from the Bayanbulak Grassland (Xinjiang, China) were used in this study.

CE was confirmed using the ultrasound technique. The anesthesia procedures were as follows: sheep were initially subject to subcutaneous injection of atropine sulfate (0.1 mg/kg), followed by intramuscular injection of Zoletil (5 mg/10 kg, VIRBAC laboratories, 06516 Carros, France) 15 min later. Subsequently, the following agents were given via intravenous injection (1 ml/10 kg): xylazine hydrochloride (batch No.: 130412, Huamu Animal Health-Care Co., Ltd. Changchun), diazepam (batch No. 14060, Jinyao Amino Acids Ltd. Co., Tianjin, China), atropine (Xixiang Changjiang Animal Pharmacy Ltd. Co., Shaanxi, China, production batch: 140201), and normal saline in a total volume of 10 mL (xylazine hydrochloride: diazepam: atropine: normal saline = 2:2:1:5). The study protocols were approved by the Ethics Committee of the First Affiliated Hospital of Xinjiang Medical University.

After anesthesia, thoracic and abdominal CT was performed (slice thickness: 5 mm) to determine the lesion number, size, and location as the baseline characteristics.

### Study design

**Fig. 1:**
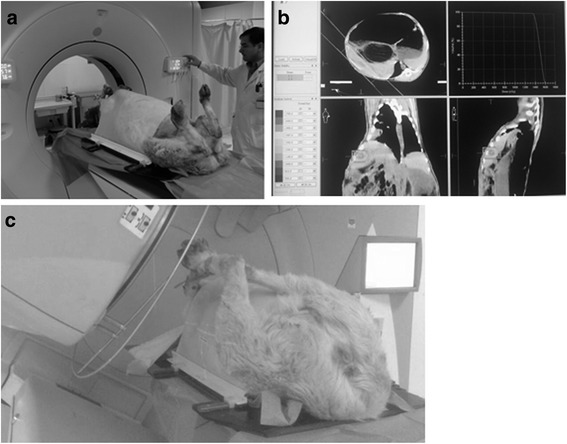
Process of radiotherapy. **a** sheep were fixed on an operative table by thermoplastic membrane; **b** radiotherapy was performed by radiotherapist with precise image-guided radiation therapy; **c** radiotherapy was performed using a linear accelerator

The sheep were randomly assigned into four groups (with five in each group), including a control group subject to no irradiation; a low-dose irradiation group subject to 30 Gy irradiation; a medium-dose irradiation group subject to 45 Gy irradiation; and a high-dose group subject to 60 Gy irradiation. After anesthesia, each sheep was fixed on an operative table by using a thermoplastic membrane. Subsequently, a computer tomography (CT) scan was performed using a 16-slice Brilliance CT Big Bore scanner (Philips, USA). The proposed irradiation shape was outlined by professional radiologists and radiotherapy was performed by experienced radiotherapists. The radiotherapy was performed in three fractions with an interval of 2 days between each fraction within 7 days to reach the predefined irradiation volume (see Fig. 1).

A routine blood test was conducted and liver function was determined before radiotherapy, as well as 3 days, 1 month, and 3 months after radiotherapy. Meanwhile, a CT scan was performed 3 months after radiotherapy to determine the changes in lesions. After radiotherapy, all sheep were followed up for 3 months to record any changes in their appetite, activity, and fur.

### Light and electron microscopy

Three months after radiotherapy, the liver was removed from each sheep to determine the changes of hydatid cyst(s) in the target lesions. The paraffin-embedded tissues were fixed with 4% paraformaldehyde. After hematoxylin and eosin staining, a light microscope was used to detect protoscolices of *E. granulosus* in the germinal layer and stratum corneum, pathological changes, and the development of cysts. Hydatid cysts were cut into 1 mm × 1 mm × 1 mm blocks, and were immediately fixed with 3% glutaraldehyde potassium oxalate (90 mmol/L) solution at 0 – 4 °C. The tissues were sliced, dehydrated by gradient ethanol and acetone, and double stained by uranyl acetate and lead citrate. Finally, the sections were observed by electron microscopy to detect ultrastructural changes.

### Real-time polymerase chain reaction (PCR)

Total RNA was extracted from the endocyst using TRIzol (batch No.: 15596026, Life, USA), followed by reverse transcription into cDNA. Real-time PCR was performed on a CFX96 Touch™ Real-Time PCR Detection System (Bio-Rad, CA, USA) using the primers described in Table 1. The mRNA level was normalized by GAPDH. The amplification results for real-time PCR were calculated according to the 2 (-ΔΔCt) method [9].

**Table 1:** Primer sequences of EgTPX, EgEPC1 and Eg HSP70

Genes	Primers (5’-3’)	Product size
EgTPX	Forward: TTTCTTAGATAAGCTCGACTCCAA Reverse: AGTATATAGACCGGTGAATTAAGGG	196bp
EgHSP70	Forward: GAGGAGTTGTGTTCGGACCT Reverse: GTCCGGGTTTATCGACTTGT	145bp
EgEPC1	Forward: TTTCTTAGATAAGCTCGACTCCAA Reverse: AGTATATAGACCGGTGAATTAAGGG	151bp
Caspase-3	Forward: CTTTGCTTGCGTCATCCTTA Reverse: GGCAGGCCTGAATAAAGAAC	152bp
Gadd45	Forward: TGGAATGCGCTCTACTATCG Reverse: GCCATGGCCTTGATAAAGAT	165bp
actin	Forward: TCAATCCTAAAGCCAATC Reverse: CGTACAACGACAGCAC	163bp

### Immunohistochemistry

Immunohistochemistry was performed using rabbit anti-transforming growth factor β (TGF-β) antibody (1:500, batch No. BS-0086R, Biosynthesis Biotechnology Co. Ltd, Beijing, China) and secondary donkey anti-rabbit antibody (1:300, batch No. ab6802, Aibokang Co. Ltd, Shanghai, China). All procedures were conducted using a commercial TGF-β kit (Yansheng Co., Ltd. Shanghai, China) according to the manufacturer’s instructions (Bioss, Beijing, China). Cytoplasm stained in a brown colour was considered as positive for TGF-β.

### Immunofluorescence

The hydrated paraffin sections were deparaffinised, endogenous peroxidase blocked, antigen retrieved, and blocked by 10% goat serum. Then, the sections were incubated with self-prepared 1% goat serum primary antibody (EPC1, 1:50) at 4 °C overnight. Afterwards, the secondary antibody (1:1 000, batch: A31571, donkey anti-mouse IgG, Alexa Fluor® 647, USA) was added and incubated at room temperature for 1 hour. After washing with PBS, DAPI solution (Solarbio, Beijing, China) (DAPI: methanol = 1:1 000) was used for staining. Finally, the images were observed under an inverted fluorescence microscope (DMI4000B, Leica, Germany).

### Statistical analysis

Data analysis was performed using SPSS 17.0 software (IBM SPSS, CA, USA). All measurement data were presented mean ± standard deviation. Analysis of variance was used to compare the groups. All *P* values of < 0.05 were considered to be statistically significant.

## Results

### Radiotherapy did not compromise the safety of the sheep

**Fig. 2:**
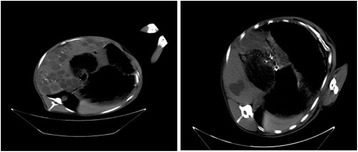
CT-diagnosed hydatid disease in sheep

All the sheep were confirmed with *E. granulosus* infection in the liver with a CT scan (see Fig. 2). No significant differences were noticed in the appetite, daily activities, and weight of the animals before and after radiotherapy (*P* > 0.05). Compared with the baseline levels, no significant differences were observed in the blood test and liver function before radiotherapy, and 3 days, 1 month, and 3 months after radiotherapy (see Tables 2 and 3). This indicates that radiotherapy did not compromise the safety of the animals.

**Table 2:** Routine blood tests before radiotherapy, 3 days, 1 month and 3 months after radiotherapy

		0 Gy	30 Gy	45 Gy	60 Gy	P
Before radiotherapy	WBC	59 ± 37	70 ± 40	80 ± 33	84 ± 56	0.163
	HB	107 ± 13	120 ± 13	104 ± 20	116 ± 9	0.331
	PLT	173 ± 56	186 ± 69	133 ± 30	238 ± 48	0.418
3 days after radiotherapy	WBC	47 ± 32	58 ± 58	35 ± 23	74 ± 57	0.327
	HB	106 ± 10	117 ± 12	106 ± 20	129 ± 13	0.079
	PLT	182 ± 42	319 ± 322	332 ± 214	477 ± 231	0.253
1 month after radiotherapy	WBC	52 ± 19	35 ± 23	54 ± 49	86 ± 46	0.267
	HB	116 ± 18	104 ± 15	104 ± 25	129 ± 10	0.149
	PLT	216 ± 50	142 ± 27	158 ± 41	308 ± 209	0.183
3 months after	WBC	60 ± 41	74 ± 57	52 ± 47	81 ± 45	0.802
	HB	111 ± 17	111 ± 8	114 ± 3	116 ± 14	0.899
	PLT	254 ± 77	130 ± 87	137 ± 54	187 ± 156	0.296

*WBC* white blood cell, *HB* hemoglobin, *PLT* platelet. There were no significant differences in WBC, HB, PLT within each group and between groups before radiotherapy, 3 days, 1 month and 3 months after radiotherapy

**Table 3:** Liver function before radiotherapy, 3 days, 1 month and 3 months after radiotherapy

		0Gy	30Gy	45Gy	60Gy	P
Before radiotherapy	ALT	207 ± 54	174 ± 33	186 ± 52	137 ± 11	0.516
	AST	64 ± 18	52 ± 14	63 ± 5	54 ± 15	0.098
	r-GT	111 ± 69	87 ± 32	76 ± 24	79 ± 25	0.381
	TP	69 ± 5	71 ± 2	72 ± 3	72 ± 5	0.832
3 days after radiotherapy	ALT	171 ± 57	134 ± 53	152 ± 57	138 ± 70	0.695
	AST	25 ± 7	18 ± 6	23 ± 12	21 ± 8	0.778
	r-GT	87 ± 32	79 ± 23	130 ± 93	117 ± 40	0.575
	TP	68 ± 7	66 ± 5	70 ± 6	64 ± 9	0.611
1 month after radiotherapy	ALT	203 ± 40	160 ± 61	145 ± 31	214 ± 71	0.019
	AST	42 ± 9	18 ± 3	22 ± 10	21 ± 10	0.227
	r-GT	76 ± 24	117 ± 40	137 ± 107	226 ± 179	0.227
	TP	68 ± 7	77 ± 6	71 ± 4	63 ± 8	0.365
3 month after radiotherapy	ALT	260 ± 113	190 ± 57	199 ± 51	136 ± 42	0.057
	AST	46 ± 20	27 ± 8	22 ± 12	23 ± 5	0.114
	r-GT	79 ± 24	195 ± 145	160 ± 98	142 ± 98	0.373
	TP	66 ± 6	68 ± 2	67 ± 3	68 ± 5	0.854

*ALT* alanine aminotransferase, *AST* aspartate aminotransferase, *r-GT* gamma-glutamyltransferase, *TP* total protein. There were no significant differences in ALT, AST, r-GT and TP within each group and between groups before radiotherapy, 3 days, 1 month and 3 months after radiotherapy

### Irradiation induced calcification in cysts in a dose-dependent manner

**Fig. 3:**
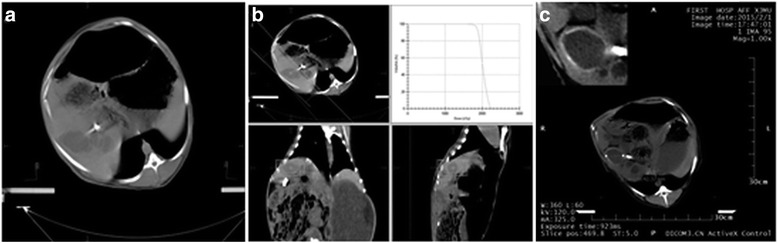
CT scan before and after radiotherapy in the high-dose group (60 Gy). **a** cyst in the right lobe of the liver before radiotherapy; **b** radiotherapy; **c** visible calcification in the cyst 3 months after radiotherapy

In the control group, no significant changes were observed in the size of the lesion at month 3 compared with the baseline level. In the low-dose group, calcification was noticed in the cysts of only one sheep, while the cysts in the other sheep showed no changes. In the medium-dose group, the irradiated cysts in four sheep remained unchanged and the size of cysts decreased in one sheep. In the high-dose group, calcification was noticed in the cysts subject to irradiation in four sheep (see Fig. 3).

### Irradiation induced cystic damages in vivo

**Fig. 4:**
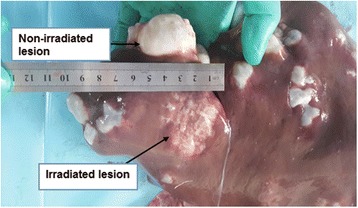
The cystic tension disappeared with the collapse in the cyst wall in the medium-dose group (45 Gy). The rear part of the irradiated liver was *dark in colour* with signs of congestion

**Fig. 5:**
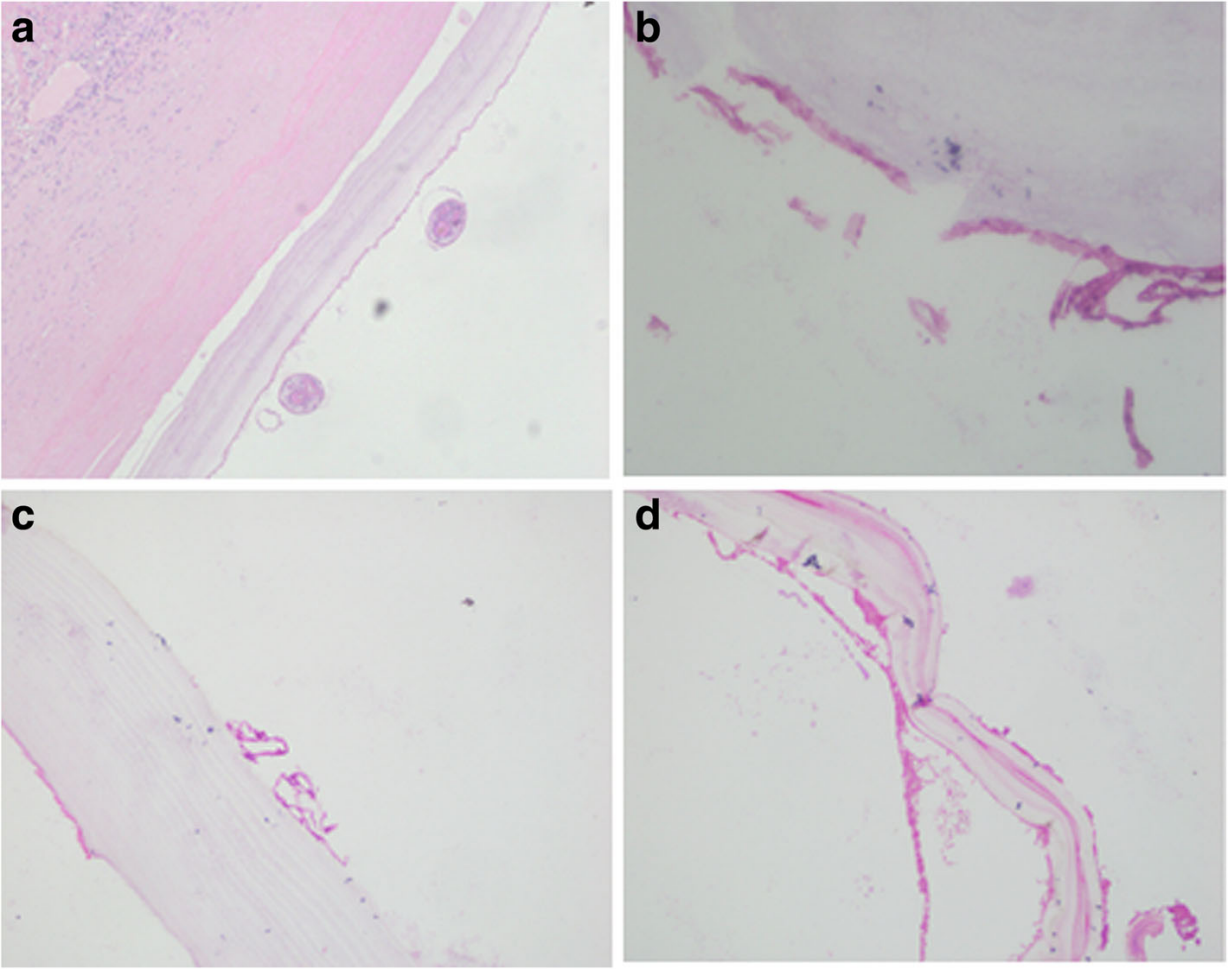
Pathology changes in the irradiated lesions (x200, under microscopy). **a** normal structure of the germinal layer in the control group; **b** atrophy in the germinal layer after irradiation in the low-dose group (30Gy); **c** separated stratum corneum and germinal layer of the irradiated cyst in the medium-dose group (45 Gy); **d** normal structure stratum corneum disappeared and the germinal layer shed off in the high-dose group (60 Gy)

In this part, pathological analysis was performed to further determine treatment efficacy. As shown in Fig. 4, in the medium-dose group, the tension of irradiated cysts was no longer present, together with the collapse of the cystic wall. However, in the non-irradiated cysts, the cystic tension was high and the cyst was filled with fluid. After irradiation, the edge of liver tissues was darkened in colour and showed signs of liver congestion. Under a light microscope, interruption was observed in the structure of the germinal layer and stratum corneum of irradiated cysts, together with universal swelling, degeneration or thinning, partial separation or breaking, karyolysis, or vanishing of germinal layer (see Fig. 5). However, cysts and protoscolex were rarely seen after irradiation. This indicates that irradiation can cause different degrees of damage to cysts.

### Irradiation induced apoptosis and necrosis in cystic cells

**Fig. 6:**
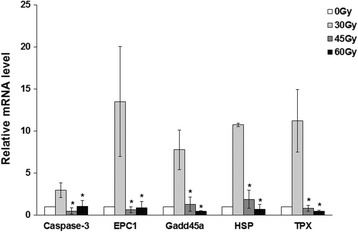
Gene expression detected by qRT-PCR. *P < 0.05, compared with the low-dose group

To investigate the molecular mechanism on the treatment efficacy of irradiation on cysts, the expressions of five key genes — namely thioredoxin peroxidase (TPX), heat shock protein 70 (HSP70), EPC1, caspase-3, and growth arrest and DNA damage-inducible protein 45 (Gadd45) — involved in cyst development were determined. The expressions of TPX and HSP70 showed a significant decrease in a dose-dependent manner (*P* < 0.05, see Fig. 6). The expression of EPC1 decreased in the medium- and high- dose groups compared with the low-dose group (*P* < 0.05). On this basis, we speculated that elevation of irradiation density inhibited the activity of cysts. Meanwhile, the expression of radiation-related apoptosis genes caspase-3 and Gadd45 decreased as the irradiation dose increased, indicating apoptosis and necrosis occurs in cystic cells after irradiation.

### Irradiation induced damages of germinal layer and cystic wall

**Fig. 7:**
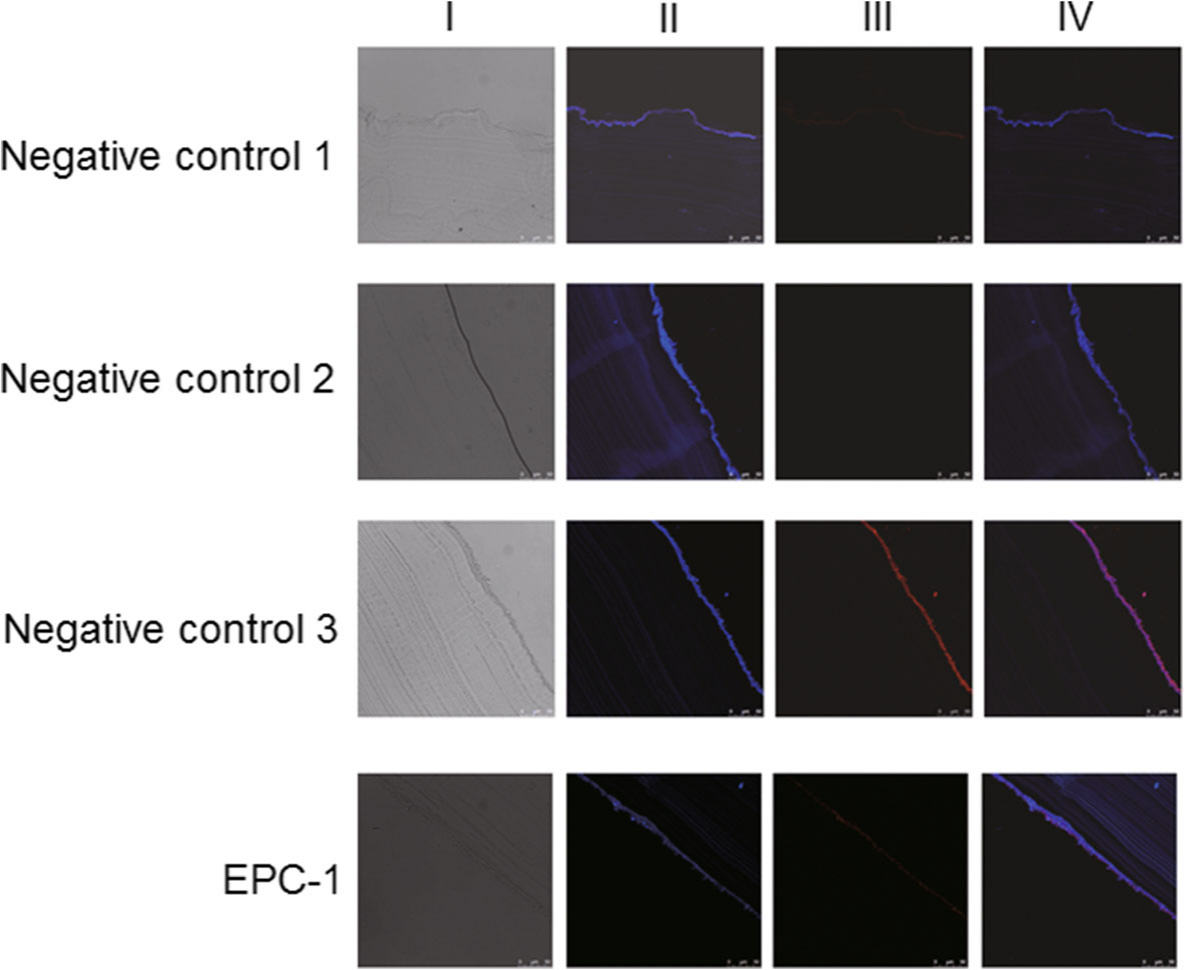
Process of indirect immunofluorescence staining. Column I: phase-contrast images; Column II: nuclear staining images (4’, 6-diamidino-2-phenylindole dihydrochloride, DAPI); Column III: anti-EPC1 fluorescence images; Column IV: nuclear staining (*blue*) image merged with anti-EPC1 fluorescence (*red*)

Murine EPC1 polyclonal antibody and Alexa Fluor® 647-labeled donkey anti-mouse IgG secondary antibody were used for indirect immunofluorescence staining. Expression of EPC1 was identified in non-irradiated tissue mainly distributed linearly along the germinal layer, while no expression was noticed in the stratum corneum (see Fig. 7). The expression of EPC1 in the irradiated cyst wall decreased and was intermittently expressed in the part near the damaged germinal layer. No expression of EPC1 was observed in the stratum corneum. In the negative control groups, no or very small non-specific scattered fluorescence reactions were observed. Taken together, we conclude that the cystic germinal layer and cystic wall were severely damaged by radiation.

### Irradiation attenuated hydatid inflammation and infection in sheep

**Fig. 8:**
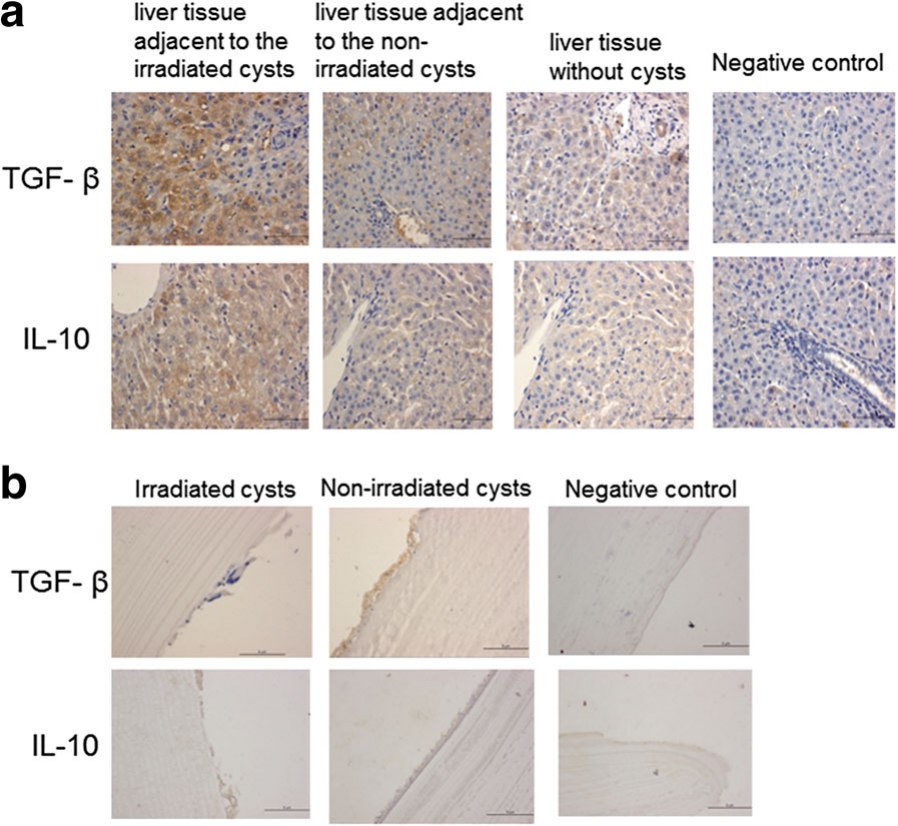
Analysis of TGF-β and IL-10 expressions (×400, under microscopy). TGF-β and IL-10 expression levels in liver tissues (**a**) and cysts (**b**) were detected by immunohistochemistry. Representative images were shown

To determine the immunological effects of radiation, immunohistochemistry was performed. The expressions of TGF-β and IL-10 were significantly upregulated in the liver tissues adjacent to the irradiated cysts. In contrast, the expressions of TGF-β and IL-10 were weak in the liver tissues adjacent to the non-irradiated cysts, or absent in the liver tissues without cysts. The germinal layer structure was incomplete in the irradiated cysts with no TGF-β and IL-10 expressions, while it was continuous with high TGF-β and IL-10 expressions in the non-irradiated cysts (see Fig. 8). This indicated that hydatid inflammation and host infection was attenuated after radiotherapy.

## Discussion

Hydatid disease can infect many mammals including sheep and humans [10, 11]. In human cystic hydatid disease, liver is the most commonly affected organ [12, 13]. In this study, sheep naturally infected with *E. granulosus* were used to mimic human infection to investigate the efficacy and safety of radiotherapy in vivo.

Radiotherapy is commonly used to treat cancers [14]. To date, radiotherapy has been used to treat the majority of malignant tumors and more than half of cancer patients would depend on or involve radiotherapy [15]. In addition, radiotherapy can also be used in benign disease treatment, such as scars, hemangioma, genital warts, tinea capitis, or abscess around the hair follicles on the head [16].

To our best knowledge, only a few clinical studies have been conducted focusing on the efficacy of radiotherapy for treating hydatid disease, mainly carried out in vitro or on rat models [7, 17]. For example, in 1998, Schmid reported a decrease of cysts after treatment using Gamma knife radiosurgery in a case with cerebral alveolar hydatid disease [18]. Zhou et al. [19] were the first to report that heavy-ion radiotherapy was more superior to X-ray in terms of inhibiting the growth of hydatid cysts.

The maximum dose of radiotherapy should be within the tolerance of normal tissue. As previously described, a dose of 30 – 60 Gy/3f is effective with safety [20, 21]. In this study, the irradiation dose ranged from 0 to 60 Gy. In the three-month follow-up, no significant differences were noticed in the appetite, daily activity, fur, and body weight of the sheep. In addition, no significant differences were observed in the routine blood and liver function tests within each group and among all groups before radiotherapy, as well as three days, one month, and three months after radiotherapy. This indicates that a dose of 30 – 60 Gy/3f induced no bone marrow suppression and liver damage in the sheep. Although the rear part of the irradiated liver showed signs of congestion, liver function remained normal due to the high decompensation capacity of the liver. Therefore, the doses adopted in this study are safe for radiotherapy to treat hepatic hydatid disease.

A CT scan is considered to be an effective way to determine the lesion size of cysts after radiotherapy. In this study, the wall calcification was classified as CE5 according to the World Health Organization ultrasound grading after irradiation, indicating that cystic activity was rather low and did not require surgical intervention. Four sheep (80%) showed significant calcification in the high-dose group (60 Gy) compared with the low-dose group, indicating the damage of the cyst was affected by the radiation in a dose-dependent manner.

In presence of an intact germinal layer in the cyst, the cyst was filled with hydatid fluid and the cystic tension was high. Upon the destruction of the germinal layer, the tension in the cyst declined. In this study, the tension in the cyst showed obvious decrease in irradiated cysts, together with the collapse of the cystic wall, while the tension in the non-irradiated cysts was still high. Under a microscope, irradiation induced interruption of the stratum corneum and the cyst’s germinal layer. As previously described, cyst destruction is a predictor for decreased hydatid activity and vitality [22]. On this basis, the pathological changes confirmed irradiation contributed to the decline of cyst growth and reproduction, which was consistent with our previous findings [7].

To investigate the potential molecular mechanism in the radiation effects on hydatid disease, we determined the expression of related genes using real-time PCR. TPX is expressed in all developmental stages of *E. granulosus*, which is of great importance in repairing the injury induced by reactive oxygen and antagonizing the host immune cells [23]. EPC1 is polypeptide screened from the cDNA library of protoscolex of *E. granulosus* with a sensitivity of 92.2% and a specificity of 95.6% [24]. The protein is present on the surface of the germinal layer and can be used as an indicator for hydatid growth. HSP70 is highly expressed and involved in protein translation, transport, and degradation [25, 26]. In our study, the expression of these genes, together with the decrease of the oxidation resistance, growth activity, and oxygen free radicals clearance, was noticed after irradiation in a dose-dependent manner, indicating radiation can kill cysts. Meanwhile, the expression of caspase-3 and Gadd45 related to apoptosis and necrosis [27] decreased after radiation. This implied that large doses of radiation would directly cause cell apoptosis and necrosis.

To further detect EPC1’s expression before and after radiotherapy, immunofluorescence detection was carried out. Our results indicate that EPC1 was mainly continuously and linearly distributed along the germinal layer of the non-irradiated normal cyst wall, and intermittently expressed along the germinal layer of the irradiated cyst wall. No expression of EPC1 was noticed in the stratum corneum after irradiation. This indicates that EPC1 expressed in the part near the germinal layer surface and was sensitive to cyst activity. The protein expression decreased after radiotherapy, suggesting that radiotherapy contributed to the decrease of cyst activity.

It has been reported that liver hydatid disease contributes to the upregulation of TGF-β and IL-10 expressions in the host [28]. The expressions of TGF-β and IL-10 in the liver were also shown to increase after irradiation [29]. In this study, the TGF-β and IL-10 expressions were significantly higher in the liver tissue adjacent to the irradiated cysts compared with the non-irradiated liver tissue, indicating cystic infection and radiotherapy promoted the liver injury pathway mediated by TGF-β and IL-10. Meanwhile, slight expressions of TGF-β and IL-10 were identified in the liver without cysts nearby, demonstrating a tendency for the host affected by *E. granulosus* to develop liver fibrosis. Furthermore, the expressions of TGF-β and IL-10 in the cystic wall obviously decreased after irradiation, indicating that irradiation contributes to the attenuation of the inflammatory reaction and infection in the host.

Our study had some limitations. Currently, surgery and chemotherapy are commonly used for the management of hydatid disease. In the present study, we could not compare the efficiency of radiotherapy and that of chemotherapy in this study. In the future, we will focus on conducting a comparison between these treatments in order to further investigate the efficiency of radiotherapy.

## Conclusions

This study was the first to investigate the efficacy and safety of radiotherapy on sheep naturally infected by *E. granulosus*, taking into account CT scans, pathology, and molecular biology. Our results indicate that radiotherapy can be a safe option for treating hydatid disease, especially for patients who do not have opportunities for surgery or are irresponsive to chemotherapy.

## Additional file

Additional file 1. Multilingual abstracts in the five official working languages of the United Nations. (PDF 1021 kb)

## Abbreviations

CT: Computer tomography
Gadd45: Growth arrest and DNA damage inducible protein 45
HSP70: Heat shock protein 70
PCR: Polymerase chain reaction
TGF-β: Transforming growth factor β
TPX: Thioredoxin peroxidase
