# Supplementary material for: Efficacy of radiotherapy for the treatment of cystic echinococcosis in naturally infected sheep
Source: Infect Dis Poverty. 2017 May 3;6:88. doi: 10.1186/s40249-017-0301-7 (PMC5414231; doi:10.1186/s40249-017-0301-7)
Supplement: Additional file 1. — Multilingual abstracts in the five official working languages of the United Nations. (PDF 1021 kb) [file 40249_2017_301_MOESM1_ESM.pdf]

Translation of the abstract into the five official working languages of the United Nations

فعالية العلاج الإشعاعي لعلاج داء الحويصلات المائية في الأغنام المصابة بشكل طبيعي

روي ماو، ون باو تشانج، هونج تشى تشى تاو جيانج قه وو بنج-فاي لو، أبدولا إينيبير، قه شانج لين شو، جي هاو، شي شو، هاي تاو لى جون لي، سونج أن تشانج، يونج شينج باو، هاو ون

ملخص

خلفية: يستخدم العلاج الإشعاعي عادة لعلاج السرطان. حتى الآن، لم يكن هناك أي دراسة تركز على آثار العلاج الإشعاعي على داء العداري في الحيوانات الكبيرة. في هذه الدراسة، ونحن نهدف إلى تحقيق كفاءة وسلامة العلاج الإشعاعي لعلاج مرض العداري الناجم عن المشوكة الحبيبية في الأغنام المصابة بشكل طبيعي.

الطرق: تم استخدام الموجات فوق الصوتية لفحص الأغنام المصابة بشكل طبيعي في المناطق الموبوءة بالمشوكات في شينجيانج، الصين. أكد التَصْوِيرٌ المَقْطَعِيٌّ المُحَوسَب (CT) وجود حويصلاتلمائية. تم تقسيم عشرين من الأغنام المصابة بشكل طبيعي بالمُشَوَّكة الحُبَيْبِيّة في الكبد و / أو الرئتين عشوائيا إلى أربع مجموعات إحداها لا يتلقون أي إشعاع، أو إشعاع أشعة سينية منخفضة (30 جراي) ومتوسطة (45 جراي)، وجرعة عالية (60 جراي)، على التوالي. بعد ثلاثة أشهر من العلاج الإشعاعي، تم إجراء الأشعة المقطعية لقياس التغيرات في الخراجات. تم جمع أكياس الطفيليات وأنسجة كبد المضيف لتحليل الأنسجة والتعبير الجيني.

النتائج: في الحيوانات الخاضعة للإشعاع، لوحظ عدم وجود فروق كبيرة في شهيتها، والأنشطة اليومية، والوزن قبل وبعد العلاج الإشعاعي. وقد لوحظ تكلس شديد في الخراجات في الحيوانات التي تخضع لجرعة عالية من الإشعاع مقارنة مع المجموعات تخضع لجرعات منخفضة ومتوسطة. وأظهر تَلْوِيْنُ الهيماتوكسيلين-أيوزين أن التشعيع ساهم في وقوع الضرر في هيكل الكيس والنواة في الطبقات الجرثومية. أظهر تفاعل البلمرة المتسلسل الكمي أن TPX وHSP70 انخفض بشكل ملحوظ في الطريقة التي تعتمد على الجرعة (P <0.05). انخفض EPC1 في المجموعة التي تتلقى جرعة متوسطة وجرعة عالية مقارنة مع المجموعة التي تتلقى جرعة منخفضة (P <0.05). وفي الوقت نفسه، انخفضت جينات الاستماتة المرتبطة بالإشعاع caspase-3 وGadd45 عند زيادة جرعة التشعيع.

الاستنتاجات: العلاج الإشعاعي هو الخيار الأفضل من حيث الكفاءة والسلامة لعلاج داء الحويصلات المائية في الأغنام ذات الاستجابة الجزئية أو المستقر لديها المرض في الشهر 3. في المستقبل، قد يصبح تثبيط النشاط الكيسي باستخدام العلاج الإشعاعي بمثابة علاججديد لمرض العداري.

Translated from English version into Arabic by Mahmoud Sami, through

TRANSLATORS WITHOUT BORDERS

放射治疗自然感染肝细粒棘球蚴病绵羊的疗效评价

摘要

引言：放疗是肿瘤的主要治疗手段。既往尚未发现有研究以大动物为研究对象研究放疗对包虫病的安全性。本文旨在评价放疗对自然感染肝细粒棘球蚴病的绵羊的有效性与安全性。

方法：从中国新疆牧区用B超筛选20只自然感染肝细粒棘球蚴病的绵羊，经CT验证后将感染羊随机分为4组：高剂量组（*n* = 5，60 Gy）、中剂量组（*n* = 5，45 Gy）、低剂量组（*n* = 5，30 Gy）、对照组（*n* = 5，0 Gy）。放疗后三月复查CT，对比放疗前后目标病灶的变化。处理标本后取放疗区肝内细粒棘球蚴囊和宿主组织做组织学和基因表达的分析。

结果：放疗前后感染羊的食欲、一般生活状况、体重无明显变化，相对于中低剂量组，高剂

量组包囊壁钙化明显，HE 染色结果显示受照射包囊的角质层及生发层结构遭到不同程度破坏，荧光定量 PCR 检测结果显示：EgTPX 和 HSP70 表达随着剂量的升高表达降低，$P < 0.05$；EgEPC1 表达低剂量组明显高于中高剂量组，$P < 0.05$；同时，放疗相关的凋亡基因 caspase-3 and Gadd45 随着放射剂量的升高表达降低。
结论：放射治疗包虫病安全可行，三个月评价病灶达到稳定和部分缓解，分子生物学水平上包囊的活性被抑制，放射治疗有望成为包虫病治疗的新手段。

Translated from English version into Chinese by Rui Mao

Effets de la radiothérapie pour le traitement de l'échinococcose kystique chez des ovins infestés naturellement

Rui Mao, Wen-Bao Zhang, Hong-Zhi Qi, Tao Jiang, Ge Wu, Peng-Fei Lu, Abudula Ainiwaer, Ge Shang, Lin Xu, Jie Hao, Xi Shou, Hai-Tao Li, Jun Li, Song-An Zhang, Yong-Xing Bao et Hao Wen

RÉSUMÉ
Contexte : La radiothérapie est couramment employée dans le traitement des cancers. Aucune étude à ce jour ne s'est intéressée sur son intérêt contre l'hydatidose du bétail. Notre étude examine l'efficacité et l'innocuité de la radiothérapie dans le traitement de l'hydatidose causée par *Echinococcus granulosus* chez des ovins infestés naturellement.
Méthodes : Des moutons infestés naturellement ont été dépistés par échographie dans une région d'échinococcose endémique de la province du Xinjiang, en Chine. La présence de kystes hydatiques a été confirmée par tomodensitométrie (TDM). Vingt moutons infestés naturellement, présentant des kystes d'*E. granulosus* dans le foie et/ou les poumons, ont été randomisés en quatre groupes, respectivement non irradiés et traités par irradiation aux rayons X à faible (30 Gy), moyenne (45 Gy) et forte dose (60 Gy). Après trois mois de radiothérapie, un scanner a été réalisé pour mesurer l'évolution des kystes. Les kystes parasitaires hépatiques et les tissus des hôtes ont été prélevés pour examen histologique et analyse de l'expression des gènes.
Résultats : Les animaux irradiés n'ont manifesté aucun changement significatif de l'appétit, de l'activité quotidienne ou du poids après la radiothérapie. Les kystes irradiés à forte dose sont apparus fortement calcifiés, à la différence de ceux des groupes à faible et moyenne dose. La coloration à l'hématoxyline-éosine a montré que l'irradiation endommageait la structure des kystes et les noyaux des couches germinales. La RCP quantitative a démontré que l'expression de TPX et HSP70 baissait significativement, d'une manière dépendante de la dose ($P < 0,05$). L'expression d'EPC1 était diminuée dans les groupes à forte et moyenne dose par rapport au groupe à faible dose ($P < 0,05$). Cependant, l'expression des gènes caspase-3 et Gadd45 associés à l'apoptose radio-induite a diminué avec l'augmentation de la dose d'irradiation.
Conclusions : La radiothérapie est une option raisonnablement efficace et sûre de traiter l'échinococcose kystique chez les ovins, avec une réponse partielle ou une stabilisation de la maladie à 3 mois. L'inhibition de l'activité kystique par la radiothérapie pourrait constituer, à l'avenir, une nouvelle option de traitement de l'hydatidose.

Translated from English version into French by Suzanne Assenat, through

Эффективность лучевой терапии при лечении кистозного эхинококкоза у овец, зараженных в естественных условиях

Руи Мао, Вэнь-Бао Чжан, Хун-Чжи Ци, Тао Цзян, Гэ Ву Пэн-Фэй Лу, Абудула Аиниваер, Гэ Шан, Лин Сюй, Цзе Хао, Си Шу, Хай-Тао Ли, Юнь Ли, Сонг-Ан Чжан, Юн-Син Бао и Хао Вэнь

ТЕЗИСЫ

Исходный контекст. Лучевая терапия — это распространённый способ лечения рака. Исследований в области применения этого метода при лечении эхинококкоза у крупных животных пока не проводилось. В настоящей работе мы постараемся проанализировать эффективность и безопасность лучевой терапии при лечении эхинококкоза, вызванного *Echinococcus granulosus* у овец, зараженных в естественных условиях.

Методы. Для изучения овец, зараженных в естественных условиях в эндемичном очаге в Синьцзяне, Китай, применялось УЗИ. Компьютерная томография (КТ) подтвердила наличие эхинококковых кист. Двадцать овец с печенью и/или легкими, пораженными *E. Granulosus,* были рандомизировано разделены на четыре группы по степени рентгеновского облучения: без облучения, низкая доза (30 Гр), средняя (45 Гр), высокая (60 Гр). После трех месяцев лучевой терапии произвели КТ с целью выявить изменения в кистах. Были собраны печеночные паразитарные кисты и питающие ткани для гистологии и анализа экспрессии генов.

Результаты. После лучевой терапии у подверженных облучению животных не было выявлено существенных изменений в аппетите, повседневной деятельности и весе. Была отмечена сильная кальцификация в кистах животных, подверженных высокой дозе облучения, в отличие от групп со средней и малой дозой. Окраска гематоксилином и эозином показала, что облучение способствует повреждению структуры кисты и ядер клеток в герминативных оболочках. Количественная ПЦР показала, что экспрессия TPX и HSP70 значительно уменьшалась в зависимости от дозы облучения ($P < 0,05$). Экспрессия EPC1 снижалась в группах со средней и высокой дозой по сравнению с группой с низкой дозой ($P < 0,05$). Между тем, экспрессия радиационно-связанных апостозных генов каспазы-3 и Gadd45 уменьшалась с увеличением дозы облучения.

Заключения. Радиотерапия является вариантом с удовлетворительной степенью эффективности и безопасности при лечении кистозного эхинококкоза у овец: наблюдается частичная ремиссия или стабилизация заболевания на 3 месяце лечения. В будущем ингибирование кистозной активности при помощи лучевой терапии может применяться в качестве нового способа лечения эхинококкоза.

Translated from English version into Russian by Vadim Frolenko, through

Eficacia de la radioterapia en el tratamiento de equinococosis quística en ovejas naturalmente infectadas

Rui Mao, Wen-Bao Zhang, Hong-Zhi Qi, Tao Jiang, Ge Wu, Peng-Fei Lu, Abudula Ainiwaer, Ge Shang, Lin Xu, Jie Hao, Xi Shou, Hai-Tao Li, Jun Li, Song-An Zhang, Yong-Xing Bao y Hao Wen

RESUMEN

Antecedentes: La radioterapia por lo general se utiliza para tratar el cáncer. Hasta la fecha, no ha habido ningún estudio que se centre en los efectos de la radioterapia sobre la enfermedad hidatídica en animales grandes. En el presente estudio, nos propusimos investigar la eficacia y seguridad de la radioterapia para el tratamiento de la enfermedad hidatídica producida por *Echinococcus granulosus* en ovejas naturalmente infectadas.

Métodos: Se utilizó el ultrasonido para tamizar ovejas naturalmente infectadas en una zona endémica de Xinjiang, China. Una tomografía axial computada (TAC) confirmó la presencia de quistes hidatídicos. Veinte ovejas naturalmente infectadas por *E. granulosus* en el hígado y/o pulmones fueron asignadas aleatoriamente en cuatro grupos que no recibieron radiación, o una radiación X de baja (30 Gy), media (45 Gy) y alta dosis (60 Gy), respectivamente. Luego de tres meses de radioterapia, se realizó una TAC para medir los cambios en los quistes. Se recolectó tejido de los quistes parasíticos hepáticos y del huésped para llevar a cabo un análisis histológico y de expresión génica.

Resultados: En los animales que no estuvieron expuestos a radiación, no se observaron diferencias significativas en su apetito, actividades diarias y peso antes y después de la radioterapia. Se notó una severa calcificación en los quistes expuestos a altas dosis de radiación en comparación con los grupos que estuvieron expuestos a dosis bajas y medias. La tinción de hematoxilina y eosina mostró que la radiación contribuyó al daño de la estructura quística y núcleo en las capas germinales. La PCR cuantitativa demostró que la expresión de TPX y HSP70 disminuía significativamente de manera dosis-dependiente ($P < 0{,}05$). La expresión de EPC1 disminuía en los grupos con dosis medias y altas en comparación con los grupos con dosis bajas ($P < 0{,}05$). Mientras tanto, la expresión de los genes de la apoptosis relacionados con la radiación, caspasa-3 y Gadd45, disminuía con el aumento en la dosis de radiación.

Conclusiones: La radioterapia es una opción con eficacia y seguridad satisfactorias para el tratamiento de la equinococosis quística en ovejas con respuesta parcial o enfermedad estable en el tercer mes. En el futuro, la inhibición de la actividad quística mediante el uso de la radioterapia podría llegar a servir como un nuevo régimen para el tratamiento de la enfermedad hidatídica.

Translated from English version into Spanish by Maria Alejandra Aguada, through

TRANSLATORS
WITHOUT BORDERS
